# Supplementary material for: Identification and Characterization of a New Regulator, TagR, for Environmental Stress Resistance Based on the DNA Methylome of Streptomyces roseosporus
Source: Microbiol Spectr. 2023 May 8;11(3):e00380-23. doi: 10.1128/spectrum.00380-23 (PMC10269677; doi:10.1128/spectrum.00380-23)
Supplement: Supplemental file 1 — Supplemental material. Download spectrum.00380-23-s0001.pdf, PDF file, 1.3 MB [file spectrum.00380-23-s0001.pdf]

Identification and Characterization of a New Regulator TagR for Environmental Stress Resistance Based on the DNA Methylome of *Streptomyces roseosporus*

Wen-Li Gao[a,b], Jiao-Le Fang[a,b], Chen-Yang Zhu[a,b], Wei-Feng Xu[a,b], Zhong-Yuan Lyu[a,b], Xin-Ai Chan[a,b], Qing-Wei Zhao[a #], Yong-Quan Li[a,b #]

[a]First Affiliated Hospital and Institute of Pharmaceutical Biotechnology, Zhejiang University School of Medicine, Hangzhou 310058, China

[b]Zhejiang Provincial Key Laboratory for Microbial Biochemistry and Metabolic Engineering, Hangzhou 310058, China

Supplementary Materials and Methods

Media and cultural conditions

S. roseosporus strains were grown at 30 °C on medium and TSB (liquid tryptic soy broth with 5% PEG6000) was the media for *S. roseosporus*, mycelium preparation, and YEME (0.3% yeast extract, 0.3% malt extract, 0.5% tryptone, 4% glucose) was used for daptomycin production. R5 solid medium worked for sporulation, while MS solid medium was used for conjugation(1). *S. roseosporus* was cultured in TSB at 280 rpm for 36 h, and then mycelia were transferred into YEME at a 1% ratio. Starting from 72 h, every 12 h, 1‰ decanoic acid feeding medium (decanoic acid: methyl oleate =1:1) was added to the fermentation broth (YEME) to promote the synthesis of daptomycin. ISP4 solid medium was used for the generation of *S. lividans,* and TSB was the media for *S. lividans* mycelium preparation. All *E. coli* strains were cultured in LB medium at 37 °C.

Plasmids and in-frame deletion and overexpression strains construction

The fragments were amplified by KOD FX (Toyobo) with corresponding primers (F+R). The linearized vectors were generated by the digestion of selected restriction endonucleases (Thermo Scientific). Amplified fragments were inserted into linearized vectors via ClonExpress MultiS One Step Cloning Kit (Vazyme) and transformed into DH5α for further study.

Integrative plasmids of pKC1139(2) containing homologous arms of the selected genes were cloned into *E. coli ET12567/pUZ8002* separately. Then the plasmids were transformed into *S. roseosporus* via conjugation. Then the gene knock-out mutants were obtained using the in-frame deletion strategy and identified by PCR (Fig. S2) with primers listed in Table S3.

For overexpression and functional complementation, genes were cloned and integrated into plasmid pIJ8661(3) via ClonExpress II One Step Cloning Kit.

Engineered plasmids were transformed into *S. roseosporus* and *S. lividans* via conjugation and then integrated into the genome. The mutants were identified by PCR (Fig. S3A-B, S7C, and S9A) with primers listed in Table S3.

Transcriptome analysis of *S. roseosporus*

The total RNA of each sample was extracted using TRIzol Reagent /RNeasy Mini Kit (Qiagen). The total RNA of each sample was quantified and qualified by Agilent 2100/2200 Bioanalyzer (Agilent Technologies, Palo Alto, CA, USA), NanoDrop (Thermo Fisher Scientific Inc.), and 1% agarose gel. 1μg total RNA was used for the following library preparation. Next generation sequencing library preparations were constructed according to the manufacturer's protocol. The rRNA was depleted from total RNA using rRNA removal kit. The ribosomal depleted RNA was then fragmented and reverse-transcribed. First strand DNA was synthesized using ProtoScript II Reverse Transcriptase with random primers and Actinomycin D. The second-strand cDNA was synthesized using Second Strand Synthesis Enzyme Mix (including dACGTP/dUTP). The purified double-stranded cDNA by beads was then treated with End Prep Enzyme Mix to repair both ends and add a dA-tailing in one reaction, followed by a T-A ligation to add adaptors to both ends. Size selection of Adaptor-ligated DNA was then performed using beads, and fragments of ~400 bp (with the approximate insert size of 300 bp) were recovered. The dUTP-marked second strand was digested with the Uracil-Specific Excision Reagent enzyme. Each sample was then amplified by PCR using P5 and P7 primers, with both primers carrying sequences which can anneal with flow cell to perform bridge PCR and P5/P7 primer carrying index allowing for multiplexing. The PCR products were cleaned up using beads, validated using a Qsep100 (Bioptic, Taiwan, China), and quantified by Qubit3.0 Fluorometer (Invitrogen, Carlsbad, CA, USA).

Then libraries with different indices were multiplexed and loaded on an Illumina HiSeq/Novaseq instrument according to the manufacturer's instructions (Illumina, San Diego, CA, USA) or an MGI2000 instrument according to the manufacturer's instructions (MGI, Shenzhen, China). Sequencing was carried out using a 2x150 paired-end (PE) configuration; image analysis and base calling were conducted by the HiSeq Control Software (HCS) + OLB + GAPipeline-1.6 (Illumina) on the HiSeq instrument, image analysis and base calling were conducted by the NovaSeq Control Software (NCS) + OLB + GAPipeline- 1.6 (Illumina) on the NovaSeq instrument, image analysis and base calling were conducted by the Zebeacall on the MGI2000 instrument.

Transmission Electron Microscope observation

For HPF, samples were dipped into external cryoprotectant (1-hexadecene) and carefully loaded into HPF sample carriers (0.2 mm depth, 16770141 Type A, Leica). The lid (16770142 Type B, Leica) was dipped in 1-hexadecene and placed on top of the sample carrier. And the samples were frozen using an HPF device (Leica EM ICE) and freeze substituted in EM AFS2 (Leica).

For freeze substitution, samples were cryosubstituted in an EM AFS2 (Leica). The sample chamber was filled with ethyl alcohol for keeping the temperature stable. The specimen carrier was transferred into a cap containing (1%OsO_4) in acetone. Program substitution started following the table below.

step	initial temperature (°C)	final temperature (°C)	time of duration (h)
1	-108	-108	1
2	-108	-90	2
3	-90	-90	60
4	-90	-60	15
5	-60	-60	24
6	-60	-30	15
7	-30	-30	15
8	-30	-0	3

For Epon embedding, samples were washed using acetone (3 times for 30min each) at 4℃, followed by acetone (2 times for 30min each) at room temperature. And the acetone was substituted with a 7:3 mixture of acetone and Epon. Incubate for 8 h. Replace the former solution (7:3 acetone/Epon) with 3:7 acetone/Epon and incubate for 12 h. Exchange this solution (3:7 acetone/Epon) with pure Epon and incubate for 12 h. Exchange pure Epon with pure Epon for another 12h. Polymerize Epon at 60℃ in an oven for 2 days.

The polymerized resin blocks were sliced by Ultramicrotome (Leica) with a thickness of 80nm. The slices were stained with uranium dioxide acetate and lead citrate, and the ultrathin slices were observed by spirit 120kV electron microscope (Thermo Fisher).

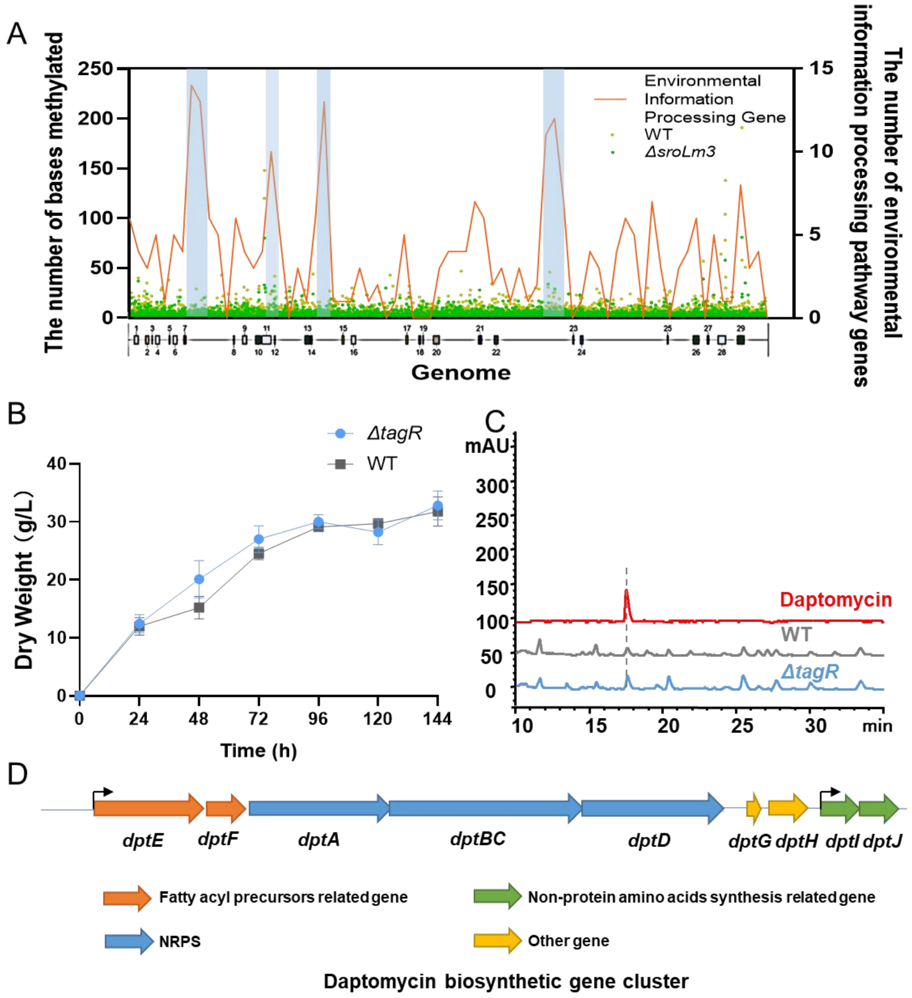

Figure S1. The fermentation experiment of daptomycin and the daptomycin biosynthetic gene cluster.

(A) Genome map of methylation modification sites and KEGG environmental information processing pathway genes. The regions marked in blue with relatively high abundance of KEGG environmental information processing pathway genes (accounting for more than 10 such genes in every 100 CDS). (B) Biomass curve of the WT and *ΔtagR* during fermentation. (C) Daptomycin yield of WT and *ΔtagR*. (D) The daptomycin biosynthetic gene cluster. Key promoters were marked with the black arrow

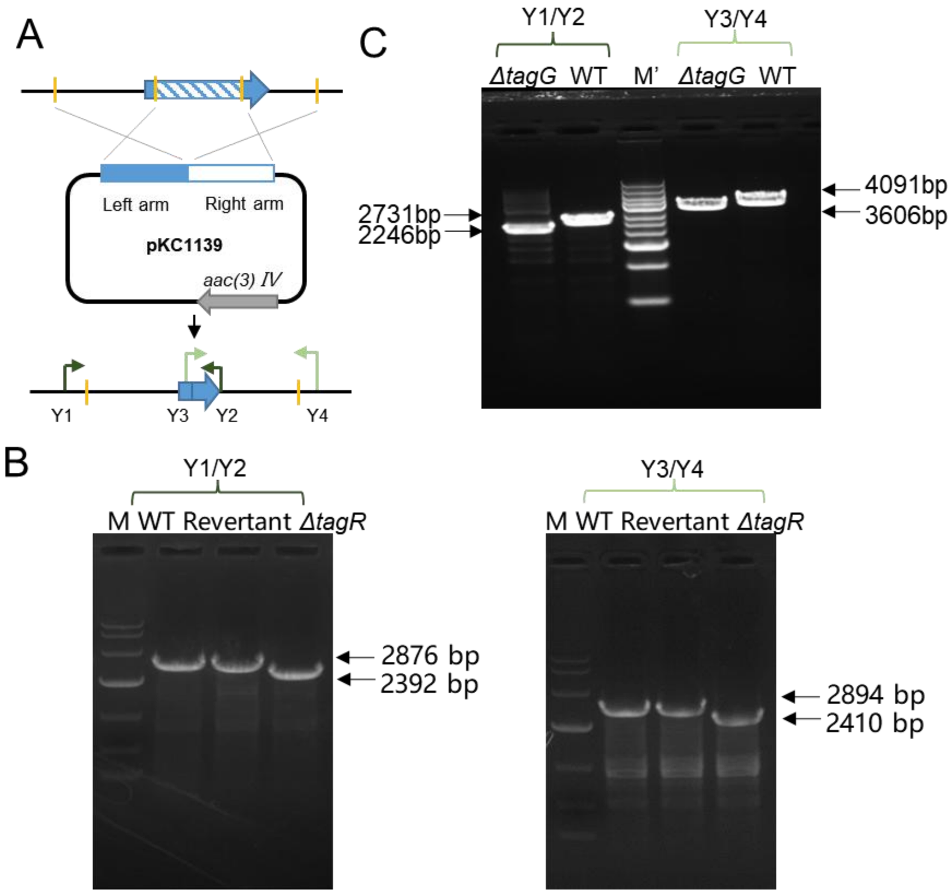

Figure S2. Schedule of in-frame deletion and identification of in-frame deletion strains.

(A) Schedule of the in-frame deletion of *tagR* and *SrtagG* in *S. roseosporus* L30. Green arrow pairs marks primer pairs used for the identification of the mutants. (B)and(C) Results of the identifications of *ΔtagR* and *ΔtagG* using PCR.M replaces for DL 10,000 DNA Marker (TaKaRa). M' replaces for 1Kb DNA Ladder (TsingKe).

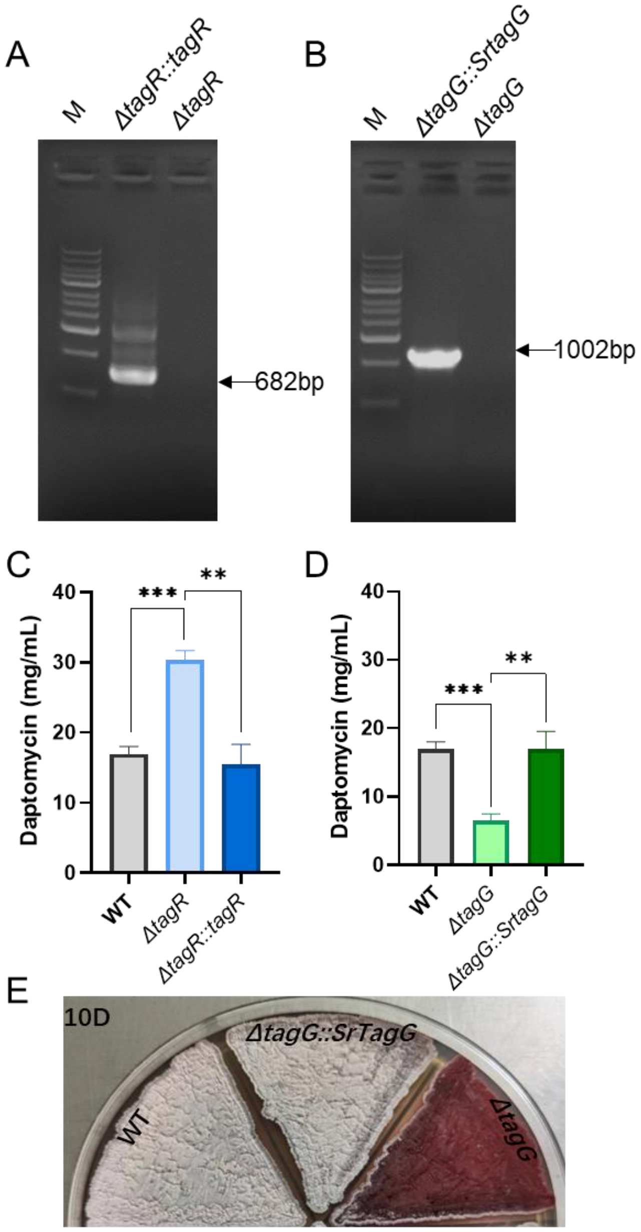

Figure S3. Identification and fermentation result of complementation strains *ΔtagR::tagR and ΔtagG::SrtagG* .

(A) and (B) Results of the identifications of *ΔtagR::tagR and ΔtagG::SrtagG*. M replaces for 1Kb DNA Ladder (TsingKe). (C) The yield of daptomycin from WT, *ΔtagR*, and *ΔtagR::tagR* fermentation experiments (n = 3, mean with SD). (D) The yield of daptomycin from WT, *ΔtagG*, and *ΔtagG::SrtagG* fermentation experiments (n = 3, mean with SD). (E) Growth status of WT, *ΔtagG*, and *ΔtagG::SrtagG*.

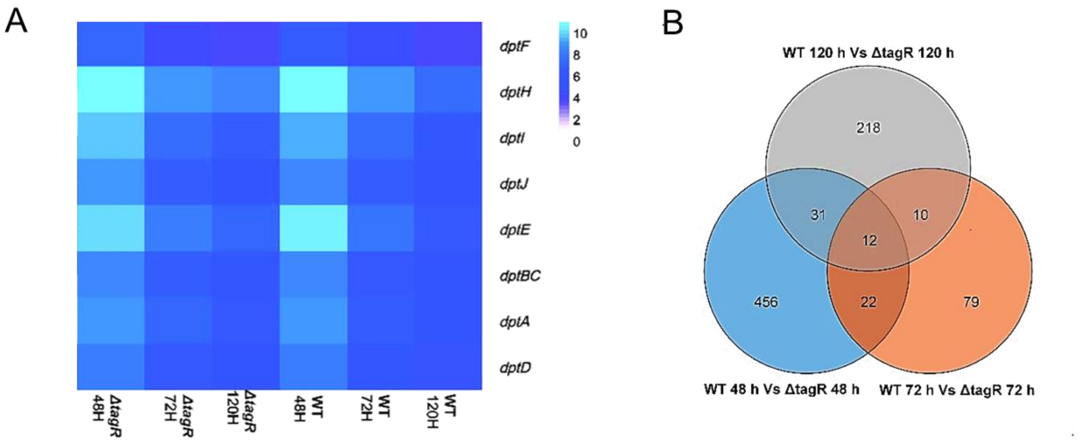

Figure S4. The transcriptome analysis of WT and *ΔtagR*

(A) Hot map of daptomycin synthesis gene cluster expression of WT and *ΔtagR* at each time point.

(B) Venn diagram of differentially expressed genes at each time point.

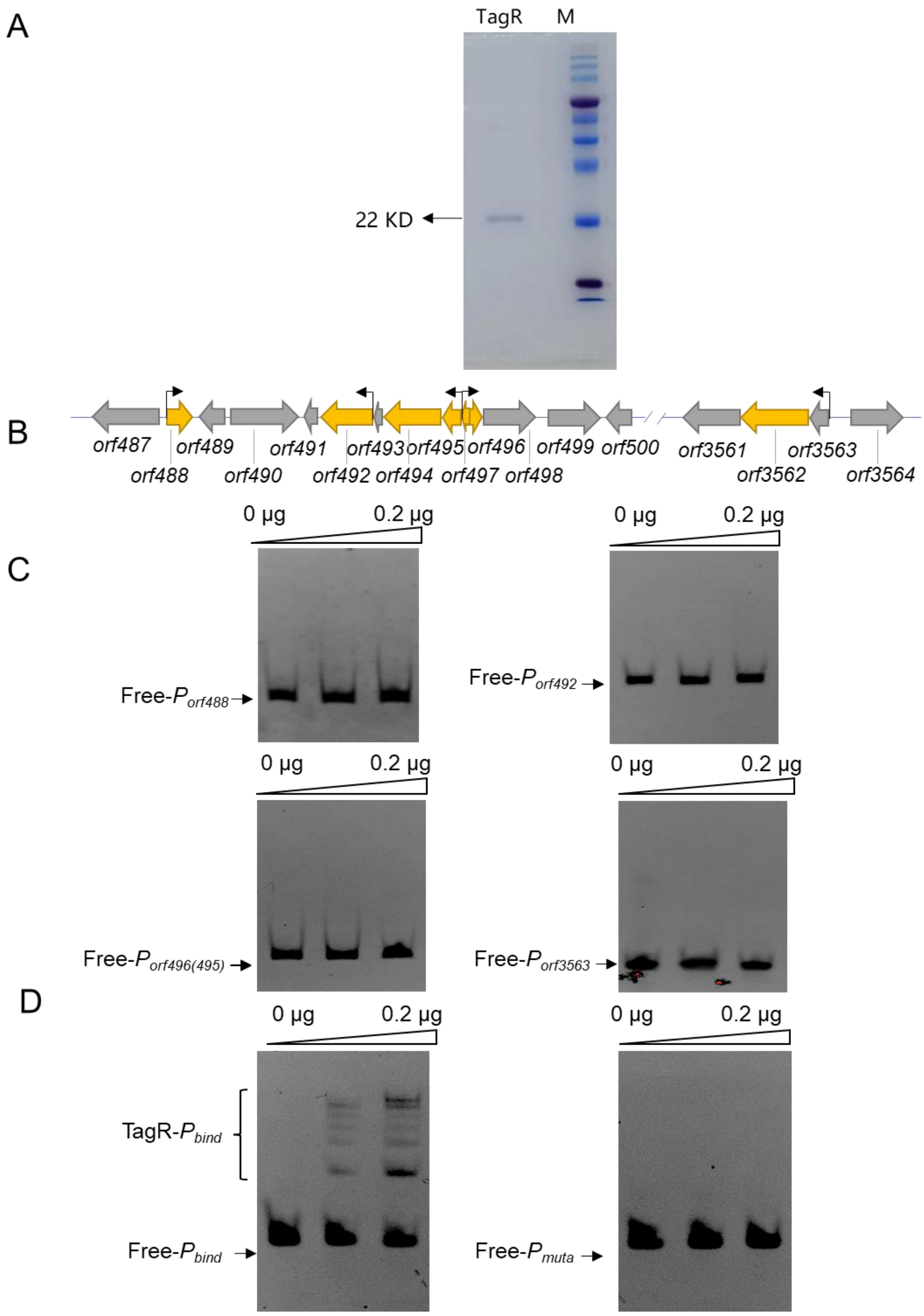

Figure S5. EMSAs and mutational analysis of the TagR-binding sites.

(A) Purified TagR protein. M replaces to 10-170 KDa Prestained Protein Ladder (Thermo). (B) Map of the differentially expressed genes (highlighted in yellow) on the genome. Promoters to which TagR is presumed to bind directly are marked with black arrows. The promoter region of *orf497* overlaps with the *orf496* CDS region, and transcription factors mostly bind in the non-coding region, so this promoter was not selected for detection. (C) EMSAs using labeled probes and the TagR protein. (D) Mutational analysis of the TagR-binding sites.

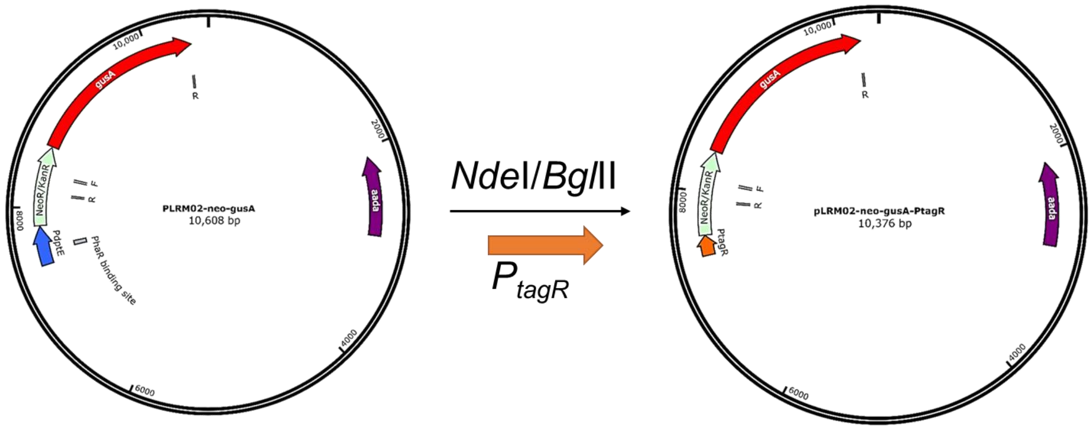

Figure S6. Construction of pRM02-*neo*-*gusA*-P_{tagR}.

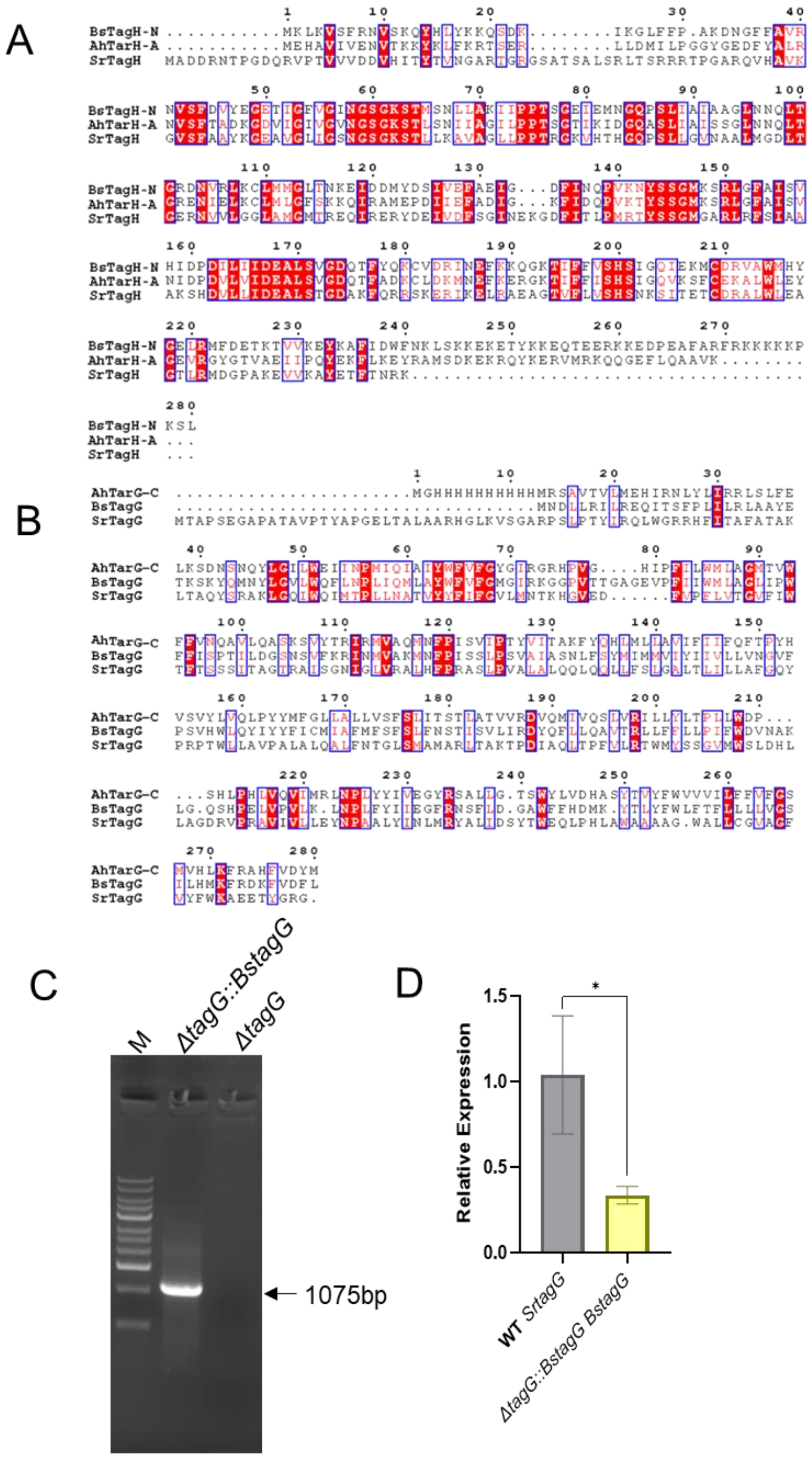

Figure S7. Homology alignment of SrTagH and SrTagG and complementation of *SrtagG*

(A) and (B) Homology alignment of SrTagH and SrTagG. (C) Results of the identifications of *ΔtagG::BstagG*. M replaces for 1Kb DNA Ladder (TsingKe). (D) Relative expression of *SrtagG* in WT and *BstagG* in *ΔtagG::BstagG*.

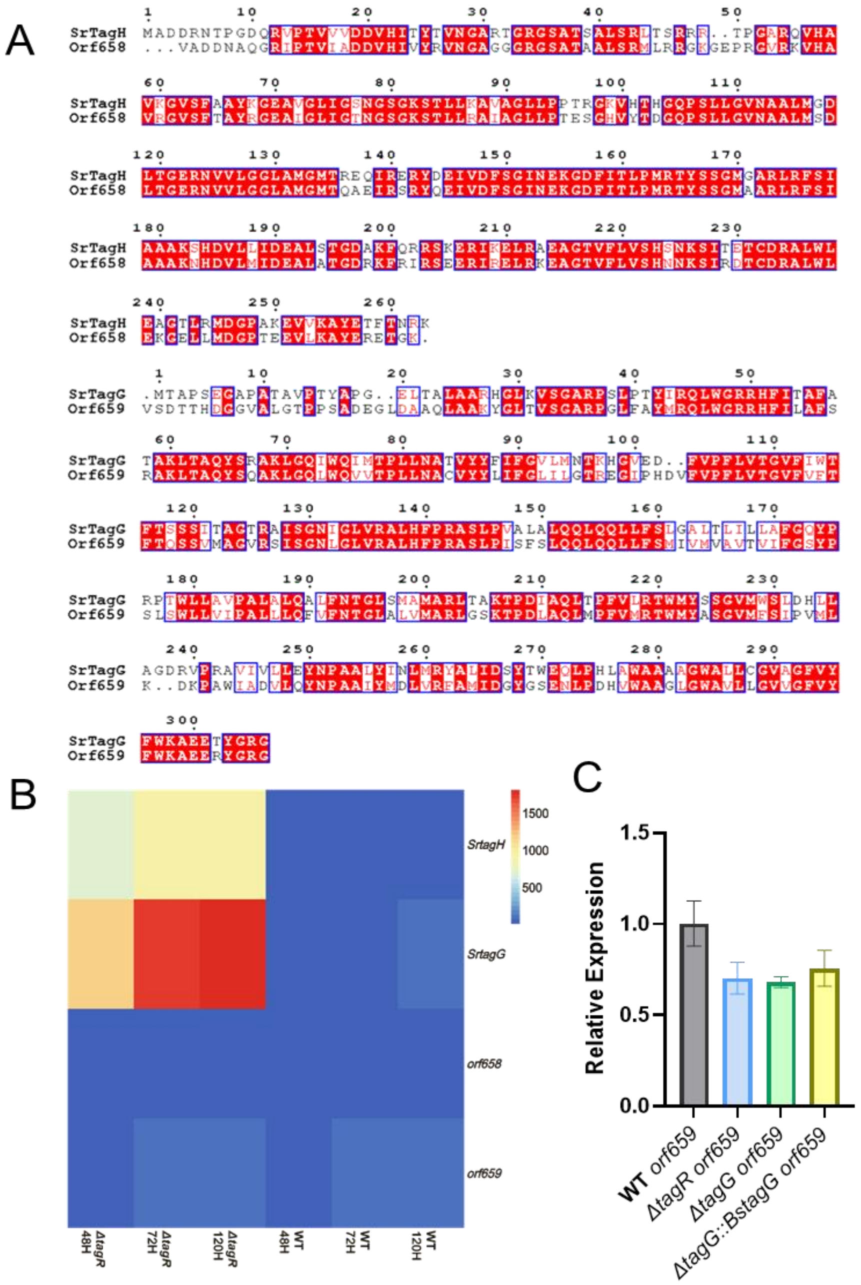

Figure S8. Alleles of SrTagH and SrTagG and their relative expressions

(A) Homology alignment of the alleles. (B) Transcriptome data of the alleles. (C) Relative expression of *orf659* in WT*, ΔtagR, ΔtagG,* and *ΔtagG::BstagG.*

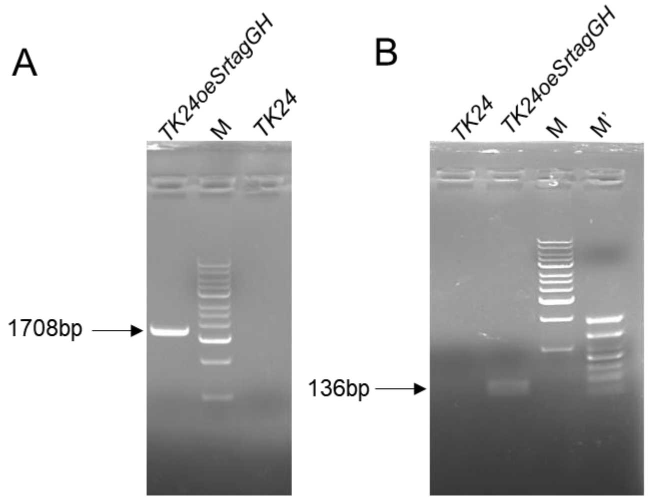

Figure S9. Identification and expression analysis of *TK24oeSrtagGH* and *TK24.*

(A) Results of the identification of *TK24oeSrtagGH* and *TK24*. (B) Results of the qPCR of *TK24oeSrtagGH* and *TK24*. M replaces for 1Kb DNA Ladder (TsingKe). M’ replaces for DL 10,000 DNA Marker (TaKaRa).

Table S1. All strains used in this study.

Strains	Genotype and characters	Sources/reference
WT	wild type*, Streptomyces roseosporus* L30, daptomycin producer	China Center for Type Culture Collection (CCTCC) No. M2010136
ΔsroLm3	In-frame deletion of *sroLm3* in WT	(4)
ΔtagR	In-frame deletion of *tagR(orf4759)* in WT	This work.
ΔtagG	In-frame deletion of *SrtagG(orf4758)* in WT	This work
ΔtagR::tagR	*tagR* cloned into pIJ8661, expressed in *ΔtagR*	This work
ΔtagG::SrtagG	*SrtagG* cloned into pIJ8661, expressed in *ΔtagG*	This work
B. s. 168	*Bacillus subtilis subsp. subtilis str.* 168	This lab
ΔtagG::BstagG	*B. s.* 168 *tagG (BstagG)* cloned into pIJ8661, expressed in *ΔtagG*	This work
WT/*gusA*	pRM02-*neo*-*gusA*-P_{tagR} conjugation into WT	This work
ΔtagR/*gusA*	pRM02-*neo*-*gusA*-P_{tagR} conjugation into *ΔtagR*	This work
ΔsroLm3ΔtagR	In-frame deletion of *sroLm3* and *tagR* in wild type, L33- *Δ orf4759*	(4)
ΔsroLm3ΔtagR/*gusA*	pRM02-*neo*-*gusA*-P_{tagR} conjugation into *ΔsroLm3/ΔtagR*	This work
TK24	*Streptomyces lividans* TK24	This lab
TK24 oeSrtagGH	*Streptomyces roseosporus* L30 *tagG* and *tagH* (*SrtagG and SrtagH*) cloned into pIJ8661, overexpressed in *TK24*	This work
E. coli DH5α	Host for general cloning	Novagen®
ET12567/pUZ8002	Conjugation host	This lab
BL21 (DE3)	Expression host for regulator TagR	This lab

Table S2. All plasmids used in this study.

Plasmids	Description	Reference
**pIJ8661**	Overexpression vector containing a strong promoter *ermEp**, integrative in *Streptomyces*	(3)
**pKC1139**	Temperature-sensitive shuttle vector for gene knock-out in *Streptomyces*	(2)
**pET28a**	Bacterial vector for protein purification	Laboratory
**pKC1139-*ΔtagR***	pKC1139 with homologous arms of *tagR*	This study
**pKC1139-*ΔtagG***	pKC1139 with homologous arms of *SrtagG*	This study
**pKC1139-$P_{tagR(tagG)}$**	pKC1139 with the promoter of *tagR* (shared with *SrtagG*)	This study
**pKC1139-P_{orf492}**	pKC1139 with the promoter of *orf492*	This study
**pKC1139-P_{orf488}**	pKC1139 with the promoter of *orf488*	This study
**pKC1139-$P_{orf496(orf495)}$**	pKC1139 with the promoter of *orf496* (shared with orf495)	This study
**pKC1139-$P_{orf3563}$**	pKC1139 with the promoter of *orf3563*	This study
**pIJ8661-*tagR***	Complementation plasmid with *tagR*	This study
**pIJ8661-*SrtagG***	Complementation plasmid with *SrtagG*	This study
**pIJ8661-*BstagG***	Overexpression plasmid with *BstagG*	This study
**pIJ8661-*SrtagGH***	Overexpression plasmid with *SrtagG and SrtagH*	This study
**pET28a-*tagR***	Protein expression vector for *tagR*	This study
**pIJ776**	amplify *neo* fragment	Laboratory
**pSET152-Ep-*gusA***	amplify the *gusA* fragment	(5)
**pLRM02-*neo-gusA***	GusA reporter plasmid with P_{dptE}	This study
**pRM02-*neo-gusA*-P_{tagR}**	GusA reporter plasmid with P_{tagR}	(6)

Table S3: All primers used in this study.

Primers	Sequence (5'-3')	Amplicon sizes(bp)	Description
tagR-Del-UF	GGGCTGCAGGTCGACTCTAGAGCCCTTCTCGTTGATGCCG	1599	Construction of pKC1139-*ΔtagR*
tagR-Del-UR	AGCCGCCAGTACAGCGGGTTCCCCGTTCGGTGGT		
tagR-Del-DF	CGCTGTACTGGCGGCTCGTC	1678	
tagR-Del-DR	TGATTACGAATTCGATATCGCGGCTTCTGCCTGGACTT		
tagR-Del-Y1	TGGCTGACCAGGAAGACCG	2876	Identification of pKC1139-*ΔtagR*
tagR-Del-Y2	GCCGTACTACAACGGGCTCACC		
tagR-Del-Y3	GGGGCTGCTGTCTGGCTCAA	2794	
tagR-Del-Y4	GCTTCGCCACTCCGTTGCTG		
tagG-Del-UF	ACGACGGCCAGTGCCAAGCTTGTTCGGCTACTTGCGGTTGG	1060	Construction of pKC1139-*ΔtagG*
tagG-Del-UR	TGATAATAGTGATAACTGCGGACCTGGATGTACTCG		
tagG-Del-DF	TGATAATAGTGATAAAACGCCGTGATGAAGTGCC	1890	
tagG-Del-DR	CATGATTACGAATTCGATATCGGTGAACCTGCTGCTCCTG		
tagG-Del-Y1	ATCTGGCGAATCTGTCGGTGGC	2731	Identification of pKC1139-*ΔtagG*
tagG-Del-Y2	ACAGCGGTCCGACGATGAGG		
tagG-Del-Y3	TCGGCTACTTGCGGTTGGT	4091	
tagG-Del-Y4	GCGGCTTCTGCCTGGACTT		
tagR-OE-F	CCAAAGGAGGCGGACATATGCTGCGGGCCATGACCA	682	Construction of pIJ8661-*tagR*
tagR-OE-R	AGAAGATCGATGTGATATCGATTATCAGGCGCGCTGAGGT		
SrtagG-OE-F	CCAAAGGAGGCGGACATATGACCGCCCCCTCGGAG	1002	Construction of pIJ8661-*SrtagG*
SrtagG-OE-R	AGAAGATCGATGTGATATCGCGCCGTTGACCGTGTA		
SrtagGH-OE-F	CCAAAGGAGGCGGACATATGACCGCCCCCTCGGAG	1708	Construction of pIJ8661-*SrtagGH*
SrtagGH-OE-R	AGAAGATCGATGTGATATCTCGGCTACTTGCGGTTGG		
BstagG-OE-F	CCAAAGGAGGCGGACATATGAATGATTTGTTGCGTATACTCA	1075	Construction of pIJ8661-*BstagG*
BstagG-OE-R	AGAAGATCGATGTGATATCTAGCCAGCAGGTTAGACATGGTC		
tagR-pro-F	TAAGAAGGAGATATACCATGCTGCGGGCCATGACCA	682	Construction of pET28a-*tagR*
tagR-pro-R	TTGTCGACGGAGCTCGAATTCGATTATC		

Primers	Sequence (5'-3')	Amplicon sizes(bp)	Description
	AGGCGCGCTGAGGT		
qRT-*hrdB*-F	GGTCCTGGTCACGGTGGTCTT	93	*hrdB* qRT-PCR
qRT-*hrdB*-R	ACCAGATTCCGCCAACCCAGT		
qRT-*dptE*-F	CTGCACGAAGGGAATCTCCA	123	*dptE* qRT-PCR
qRT-*dptE*-R	GCTCTCAAGTTCCGGGTGTT		
qRT-*dptI*-F	CGCGTACGACCACTTCAC	157	*dptI* qRT-PCR
qRT-*dptI*-R	CGAGGTCATGTAGGAGACGA		
qRT-*dptJ*-F	CTGCCGGAGATCTTCGAACT	138	*dptJ* qRT-PCR
qRT-*dptJ*-R	GAACTCGATCTCCCGGTACT		
qRT-*SrtagG*-F	GCGCCGTTGACCGTGTA	136	*SrtagG* qRT-PCR
qRT-*SrtagG*-R	GGTGGCGGGATTCGTGT		
qRT-*orf659*-F	AGGGCGGGAATCACCA	179	*orf659* qRT-PCR
qRT-*orf659*-R	TCGATCTCCGGCAACC		
qRT-*BstagG*-F	CCCCATACCAAATACGAAC	111	*BstagG* qRT-PCR
qRT-*BstagG*-R	GCGGCTTATGAAACCAAGT		
EMSA1139-F	GCACAGATGCGTAAGGAG	278	pKC1139-P_{col}
EMSA1139-R	CAGGAAACAGCTATGACATG		
tagGR-P-F	ACGACGGCCAGTGCCAAGCTTCGGGGGCGGGGCTGCTG	182	Construction of pKC1139-P_{tagR}
tagGR-P-R	CATGATTACGAATTCGATATCAGTCGGCCGCGACGAAACG		
Orf492-P-F	ACGACGGCCAGTGCCAAGCTTCAGGCCGTCTCCGTAGCG	142	Construction of pKC1139-P_{orf492}
Orf492-P-R	CATGATTACGAATTCGATATCGCTGATGCACTACCTCGGCTC		
Orf488-P-F	ACGACGGCCAGTGCCAAGCTTTCCATGGAGAGGCGGCAG	115	Construction of pKC1139-P_{orf488}
Orf488-P-R	CATGATTACGAATTCGATATCCTCGACAACCTCAGACTTCGAC		
Orf496-P-F	ACGACGGCCAGTGCCAAGCTTGCACCCGGATTAGTTCAGTGTC	129	Construction of pKC1139-$P_{orf495(orf496)}$
Orf496-P-R	CATGATTACGAATTCGATATCCAGGAGGTCTCTCTCGTCAACC		
Orf3523-P-F	ACGACGGCCAGTGCCAAGCTTGCACTGATACTCTCACATCACCGCC	77	Construction of pKC1139-$P_{orf3523}$
Orf3523-P-R	CATGATTACGAATTCGATATCGGCGGAACCCTGCCGC		
tagGR-P-binding-F(5'-FAM)	GCGGGACGGTACGGTCCCGTGGGGACGCCCTCGTTGGGACGGGACCGTATCGTCGTAACGCCGA	113	Binding site mutation assay-*Pbind*
tagGR-P-binding-R	AGTCGGCCGCGACGAAACGGTTGCGTTGCGACGTGGAAGCGCCCTACTCTCGGCGTTAC		
tagGR-P-muta-F(5'-FAM)	GCGGGACGGAAAAAAAAAAAAAAAAAAAAAAAAAAAAAAAAAAAAAAAAAAAAAAAAAAAAAAAAAAAAAAAAAAAAAGCGCTTCC	113	Binding site mutation assay-P_{muta}

Primers	Sequence (5'-3')	Amplicon sizes(bp)	Description
	ACGTCGCA		
tagGR-P-muta-R	AGTCGGCCGCGACGATTTTTTTTTTTTGCGACGTGGAAGCGC		
pIJ776-Neo-F	ATGTCATGATTGAACAAGATGGATT	796	Construction of pLRM02-*neo-gusA*
pIJ776-Neo-R	GGCATCTCAGAAGAACTCGT		
pSET152-Ep-gusA-F	CGAGTTCTTCTGAGCGGCCGCATGCTGCGGCCCGTCGAAACCC	1851	Construction of pLRM02-*neo-gusA*
pSET152-Ep-gusA-R	CTGCAGCCGGGCGGCCGCTCACTGCTTCCCGCCCTG		
gusA-*tagGR*-P-F	AACTTTAGATCCTCGAGATCTCGGGGGCGGGGCTGCTG	182	Construction of pLRM02-*neo-gusA*-P_{tagR}
gusA-*tagGR*-P-R	ATCTTGTTCAATCATCATATGAGTCGGCCGCGACGAAACGG		

Table S4 Method used in HPLC assay.

Mobile phase	A	ddH_2O with 0.1% formic acid
	B	acetonitrile
Column	Zorbax 300SB-C18, 150/4.6 mm (Agilent Technologies)	
Flow Rate	1.0 mL/min	
Wavelength	215 nm	
Column Temp	37 ℃	
Injection Volume	50 µL	
Gradient	time (min)	mobile phase A: B(v/v)
	0	90:10
	5	65:35
	55	45:55
	60	5:95
	63	90:10
	65	90:10

1. Kieser T, Bibb MJ, Chater KF, Butter M, Hopwood D, Bittner ML, Buttner MJ, editors. Practical Streptomyces Genetics: A Laboratory Manual2000.
2. Bierman M, Logan R, O'Brien K, Seno ET, Nagaraja Rao R, Schoner BE. 1992. Plasmid cloning vectors for the conjugal transfer of DNA from Escherichia coli to Streptomyces spp. Gene 116:43-49.
3. Liu S-P, Yuan P-H, Wang Y-Y, Liu X-F, Zhou Z-X, Bu Q-t, Yu P, Jiang H, Li Y-Q. 2015. Generation of the natamycin analogs by gene engineering of natamycin biosynthetic genes in Streptomyces chattanoogensis L10. Microbiological Research 173:25-33.
4. Fang J-L, Gao W-L, Xu W-F, Lyu Z-Y, Ma L, Luo S, Chen X-A, Mao X-M, Li Y-Q. 2022. m4C DNA methylation regulates biosynthesis of daptomycin in Streptomyces roseosporus L30. Synth Syst Biotechnol 7:1013-1023.
5. Guan H, Li Y, Zheng J, Liu N, Zhang J, Tan H. 2019. Important role of a LAL regulator StaR in the staurosporine biosynthesis and high-production of Streptomyces fradiae CGMCC 4.576. Science China Life Sciences 62:1638-1654.
6. Luo S, Chen X-A, Mao X-M, Li Y-Q. 2018. Transposon-based identification of a negative regulator for the antibiotic hyper-production in Streptomyces. Applied Microbiology and Biotechnology 102:6581-6592.
